# A step closer to understanding how a diet high in simple carbohydrates may cause dysbiosis

**DOI:** 10.1172/JCI180001

**Published:** 2024-05-01

**Authors:** Shrinivas Bishu, John Y. Kao

**Affiliations:** Department of Internal Medicine, Division of Gastroenterology and Hepatology, Michigan Medicine, University of Michigan, Ann Arbor, Michigan, USA.

## Abstract

The gut microbiota is an integral part of the human metaorganism that is required to shape physiologic host immune responses including host defense against pathogens. Disease-associated gut dysbiosis has been characterized by blooms of pathobionts, which are bacterial species that can drive disease under certain conditions. Pathobionts like *Enterobacteriaceae* often bloom during flares of inflammatory bowel disease (IBD) and are causally linked with IBD in murine models. In this issue of the *JCI*, Hecht and colleagues investigated how simple carbohydrates are causally linked to the bloom of the gut pathobiont *Klebsiella pneumoniae,* which belong to the *Enterobacteriaceae* family. Notably, the presence of fiber reduced the dissemination of *K*. *pneumoniae* into the blood and liver in a colitis model. Their findings provide a diet-related mechanism for gut dysbiosis, which has implications in the management of IBD and other conditions in which gut dysbiosis is an underlying factor.

## Pathobiont blooms

The human intestine is estimated to comprise 10^13^ total bacteria and encompasses over 1,000 individual species (1, 2). The gut microbiota is an integral part of the human metaorganism that is required to shape physiologic host immune responses, including protective responses to pathogens, and to regulate the balance of nutrients available to the host (3). Although the gut microbiota are largely symbiotic and protective, many chronic human diseases are linked to an altered distribution and relative abundance of gut bacteria, termed dysbiosis (4). Moreover, these dysbiotic states are often typified by blooms of pathobionts, which are bacterial species that can drive disease under certain conditions (5). Pathobionts such as *Enterobacteriaceae* often bloom during flares of inflammatory bowel disease (IBD) and are causally linked with IBD in murine models (6, 7). Thus, understanding the mechanisms regulating the expansion of and colonization resistance to pathobionts is critically important.

**Figure 1:**
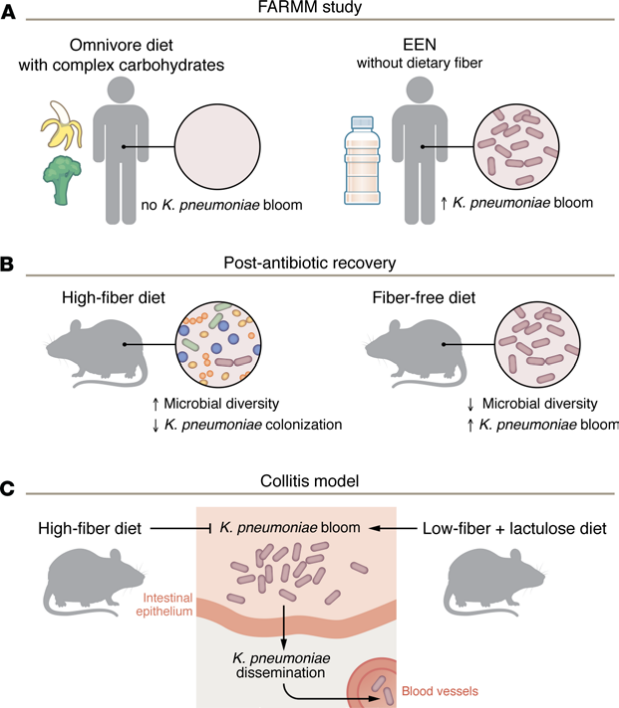
Dietary fiber regulates *K*. *pneumoniae* colonization and dissemination. (**A**) The FARMM study revealed omnivores randomized to an EEN diet, which lacks fiber, were more likely to develop a *K*. *pneumoniae* bloom compared with individuals fed a standard omnivore diet that contained complex carbohydrates. The correlation suggests that the carbon-restricting condition limited *K*. *pneumoniae* growth. (**B**) Mice fed a low-fiber diet following antibiotic treatment show reduced commensal diversity and *K. pneumoniae* colonization compared with those fed a HF diet. (**C**) Mice exposed to DSS to induce colitis are susceptible to *K*. *pneumoniae* colonization and dissemination under low-fiber dietary conditions that include lactulose, a simple carbohydrate. In contrast, a HF diet protects mice from colitis-induced dissemination.

In this issue of the *JCI*, Hecht et al. show that dietary fiber intake is an important pathway for the colonization resistance against the gut pathobiont *Klebsiella pneumoniae,* a member of the *Enterobacteriaceae* family (8). The present study dovetails off their prior Food and Resulting Microbial Metabolites (FARMM) study, which assessed the effect of dietary fiber on the recovery of the gut microbiota following ecological stress in humans (9). Participants in the FARMM study were randomized to an omnivore diet replete with complex carbohydrates or were placed on exclusive enteral nutrition (EEN), which lacks dietary fiber. Individuals on EEN exhibited marked blooms of *K*. *pneumoniae* relative to diets replete with dietary fiber (Figure 1A) (9).

*K. pneumoniae* rely on environmental nitrogen and carbohydrates as a source of energy and for growth. Moreover, *K*. *pneumoniae* utilize urease to metabolize environmental amino acids and urea into ammonia, thereby regulating their available environmental nitrogen balance. To assess the importance of nitrogen in the observed blooms of *K*. *pneumoniae*, Hecht and colleagues generated strains of *K*. *pneumoniae* that lacked urease (Δurease strain) or a key component of the nitrogen-scavenging system (Δ*ntrC* strain), thereby disrupting the nitrogen balance. Additionally, in vitro and in vivo approaches including germ-free mice, surprisingly, revealed that environmental nitrogen was abundant and therefore not the limiting factor to blooms of *K*. *pneumoniae*. These findings implied that, instead of nitrogen, carbon, in the form of simple sugars, was the limiting source.

*K. pneumoniae* favor simple carbohydrates and cannot utilize complex carbohydrates. In healthy humans, the abundance of *K*. *pneumoniae* is low in the colon. Hecht and authors considered the possibility that low *K*. *pneumoniae* counts were due to a low abundance of simple carbohydrates in the colon because simple carbohydrates are mostly absorbed in the small bowel (SB). Consistent with this concept, the authors elegantly showed that growth of *K*. *pneumoniae* was substantially more robust when cultured with SB contents, which are rich in simple carbohydrates, compared with growth when cultured with cecal contents, which would largely be composed of complex carbohydrates. Furthermore, the growth of *K*. *pneumoniae* increased substantially when glucose was added to cecal contents. This finding was also reproduced in vivo using lactulose, a source of simple carbohydrates that is not metabolized by the host but is available for luminal *K*. *pneumoniae*. Closing the loop, the authors found that *Lactobacillus* and *Bifidobacterium* species were inversely associated with blooms of *K*. *pneumoniae* in mice administered lactulose and in humans in the FARMM study (8, 9).

Finally, the Hecht and colleagues linked dietary fiber with colonization resistance and downstream physiologic effects on the host. They subjected mice to a high-fiber (HF) versus a fiber-free (FF) diet coupled with *K*. *pneumoniae* colonization after antibiotic recovery or with dextran sodium sulfate (DSS) to cause epithelial disruption that mimicked colitis. Consistent with the concept that dietary fiber is critical for colonization resistance to *K*. *pneumoniae*, mice on a HF diet had more diverse gut microbial communities and lower *K*. *pneumoniae* colonization after antibiotic recovery (Figure 1B) and were substantially protected from DSS and had markedly less systemic dissemination of *K*. *pneumoniae* (Figure 1C).

Collectively, these data cleanly link the absence of dietary fiber with blooms of *K*. *pneumoniae* after the gut microbiome was disrupted by ecological stress. Data from Hecht et al. also strongly indicate that the blooms were due to the absence of complex carbohydrates, which were probably metabolized by species that were competing for colonization and had resistance to *K*. *pneumoniae*. There is, however, one contradiction of the study when incorporating clinical experiences of EEN, which has been shown to improve disease in pediatric patients with Crohn’s disease (10). Therefore, whether the *K*. *pneumoniae* bloom observed in the EEN group strongly contributed to the IBD flare requires further investigation, at least in the pediatric Crohn’s disease setting. Nevertheless, these data support the idea that dietary therapies hold the promise of being effective in targeting disease-driving pathobionts (8).

## Diet and dysbiosis in IBD

The understanding of the determinants of IBD pathogenesis, until recent years, has mostly centered around altered functions in the mucosal immune response and host defense against resident gut microbiota (11). Once considered a disease limited to the Western world, emerging epidemiological data indicate a rapidly rising incidence of IBD, especially in newly industrialized countries in Africa, Asia, and South America, including Brazil, implicating the potential contribution of environmental factors (12). Among other factors commonly associated with industrialization is the increased consumption of refined sugar (13). In an Asia-Pacific study by Ng et al. examining environmental risk factors in IBD, the investigators found that those who drink tea were less likely to develop Crohn’s disease, but frequent juice consumption was associated with a higher disease risk (13). A recent summary of prior studies examining the link between dietary carbohydrates, sugar, and sugar-sweetened beverages also found that sugar intake was associated with an increased risk of IBD (14). It has been shown in mice that added dietary sugar can lead to gut dysbiosis and induce colitis in susceptible hosts (e.g., those with IL-10 deficiency) (15). Thus, a converging concept based on the changing epidemiology of IBD in rapidly industrialized countries is the trend of increased consumption of refined sugar and also decreased dietary fiber consumption.

Hecht et al. provide a compelling argument for how such a dietary change may alter gut microbiota and lead to a bloom in pathobionts and dysbiosis. In IBD-susceptible individuals, this diet-induced dysbiosis may result in disease onset that is typically characterized by a loss of microbe-host homeostasis at the intestinal surface. Future prospective studies are needed to address whether a diet with a low simple-to-complex carbohydrate ratio could influence the gut microbial community structures and decrease disease flares in individuals with IBD. Another related question involves the observation that the risk of IBD in immigrants usually matches that of nonimmigrants in the second generation (16, 17). This effect suggests that early life dietary exposure during the establishment of one’s gut microbiota may determine one’s IBD risk and that perhaps an emphasis on a high-fiber diet in the pediatric population may beneficially shape adult gut microbiota. This notion is consistent with the observation by Vangay et al. that migration from a non-Western country to the United States is associated with loss of gut microbiome diversity and displaced native strains, both of which increase with the duration of US residence (18). Knowledge of dietary effects on the microbiome during development may lead to a greater emphasis on restricting simple carbohydrates in infants and children, as high-sugar food consumption is certainly a major problem throughout many industrialized countries and the Western world. Therefore, the study by Hecht et al. provides an important understanding of how a diet high in simple carbohydrates could lead to gut dysbiosis, which is also an underlying mechanism of several other non-IBD diseases including obesity, metabolic disorders, and gastrointestinal malignancies.

## Version 1 05/01/2024

Electronic publication
